# Short- and Long-Term Prognosis in Hemodynamically Stable Pulmonary Embolism With Unresectable or Metastatic Malignancies: The Role of Performance Status

**DOI:** 10.7759/cureus.65795

**Published:** 2024-07-30

**Authors:** Hironori Kobayashi, Yu Oyama, Sadakatsu Ikeda

**Affiliations:** 1 Medical Oncology, Kameda Medical Center, Kamogawa, JPN; 2 Precision Cancer Medicine, Kameda Medical Center, Kamogawa, JPN

**Keywords:** venous thromboembolism, short-term prognosis, long-term prognosis, advanced malignancy, metastatic malignancy, prognosis and survival, ecog performance status, pulmonary embolism (pe)

## Abstract

Background: The simplified Pulmonary Embolism Severity Index (sPESI) has limitations when evaluating acute pulmonary embolism (PE) in patients with concurrent malignancy. Despite its utility in predicting outcomes among cancer patients, the role of the Eastern Cooperative Oncology Group Performance Status (ECOG PS) in acute PE remains underexplored. This study aims to assess the prognostic significance of ECOG PS ≥ 3 on short- and long-term mortality in acute PE with malignancy, correlating it with the sPESI.

Methods and results: We retrospectively analyzed 44 hemodynamically stable acute PE patients with unresectable or metastatic malignancies ineligible for curative treatment at Kameda Medical Center, a tertiary medical facility in Japan, from April 1, 2019, to March 2, 2023. Of these patients, 16 (36.4%) had ECOG PS ≥ 3. No 30-day mortality occurred in patients with ECOG PS ≤ 2, compared to 18.8% in those with ECOG PS ≥ 3 (p = 0.04). Groups were similar in the sPESI scores, hospital-onset PE proportion, and initial treatments. Post PE diagnosis, 92.9% of ECOG PS ≤ 2 patients and 50% of ECOG PS ≥ 3 patients received chemotherapy (p = 0.002). Cox regression analysis revealed ECOG PS ≥ 3 was independently associated with increased overall survival hazard (adjusted HR = 4.0; P = 0.002).

Conclusions: ECOG PS ≥ 3 suggests a poorer short-term prognosis and independently predicts a worse long-term prognosis in hemodynamically stable acute PE patients with advanced malignancies.

## Introduction

Acute pulmonary embolism (PE) exhibits varying short-term mortality rates across different subgroups, ranging from 1% [1-4] to exceeding 50% [5,6]. The expanding use of outpatient anticoagulation therapy in selected patients is driven by the established efficacy and safety of direct oral anticoagulants (DOACs) for acute PE [4,7,8]. Simultaneously, inpatient management with unfractionated heparin remains the gold standard for initial treatment in high-risk patients. Consequently, accurate risk classification at the time of diagnosis has become increasingly pivotal for the optimal management of acute PE.

The Pulmonary Embolism Severity Index (PESI) and simplified Pulmonary Embolism Severity Index (sPESI) stand out as widely adopted logistic regression models for predicting short-term mortality in acute PE patients [1-3,9]. These models incorporate clinical variables routinely available at presentation, previously demonstrated to be associated with all-cause mortality at three months using Kaplan-Meier methods in PE patients [6]. The sPESI, designed to estimate 30-day all-cause mortality, streamlines the PESI by excluding statistically insignificant variables in smaller cohorts [2]. Comprising age, chronic comorbidities (heart failure, chronic lung disease, and cancer), and vital signs, the sPESI effectively identifies low-risk patients suitable for outpatient anticoagulation therapy [1-4].

Patients with malignancies face an increased risk of thromboembolic complications due to a hypercoagulable state [10]. Similar to the general population, acute PE in patients with malignancies can lead to diverse clinical outcomes, ranging from favorable [11] to poor [12]. However, the applicability of the sPESI as a prognostic tool is limited in cancer patients, as all are categorized into the high-risk group, surpassing one point due to cancer comorbidities, irrespective of hemodynamic stability [2,13]. Within the cancer patient cohort, there exists a subpopulation not necessitating inpatient management, emphasizing the need for an optimized risk assessment method tailored to this subgroup.

The Eastern Cooperative Oncology Group Performance Status (ECOG PS) score serves as a prevalent method for assessing functional status and physiologic reserve in cancer patients [14]. ECOG PS ≥ 3 emerges as a critical prognostic factor for patients with unresectable or metastatic malignancies, where palliative care aimed at maintaining quality of life (QOL) often supersedes systemic chemotherapy [15,16]. Recent studies propose that ECOG PS at the time of PE diagnosis could aid in stratifying the risk of death in acute PE patients with malignancies [11,17]. However, the independence of ECOG PS from sPESI as a prognostic factor in acute PE with unresectable or metastatic malignant tumors, along with the determination of an appropriate cutoff, remains unexplored. Hospitalization is typically warranted for hemodynamically unstable PE, underscoring the need for data accumulation to ascertain the appropriateness of outpatient management for hemodynamically stable PE.

Therefore, the primary objective of this study was to assess the prognostic impact of ECOG PS ≥ 3 on short- and long-term mortality in hemodynamically stable acute PE patients with unresectable or metastatic malignancies and to evaluate the independence from sPESI.

## Materials and methods

Study design and patient population

This study represents a single-center retrospective cohort investigation that encompassed patients diagnosed with hemodynamically stable acute PE at Kameda Medical Center, a tertiary medical facility in Japan, from April 1, 2019, to March 2, 2023. These patients simultaneously had unresectable or metastatic malignancies and were not deemed suitable candidates for curative chemoradiation therapy at the time of the PE diagnosis. In general, individuals with an ECOG PS score of 3 or higher are typically not considered appropriate candidates for curative surgical interventions or chemoradiation. Consequently, this study focused its inquiry on patients diagnosed with unresectable or metastatic malignant tumors. Approval for this study was obtained from the Institutional Review Board of Kameda Medical Center (No. 23-048-231106). Informed written consent from individual patients was waived, with an opt-out provision. This research adheres to the principles outlined in the Declaration of Helsinki. Inclusion criteria stipulated the presence of hemodynamically stable acute PE confirmed by definitive imaging tests (computed tomography pulmonary angiography (CTPA), high-probability ventilation/perfusion (V/Q) scan, or pulmonary angiography), concurrently coupled with unresectable or metastatic malignancies ineligible for definitive chemoradiation therapy. Exclusion criteria comprised patients who did not utilize anticoagulation or fibrinolytic agents within 24 hours of diagnosis, absence of data concerning the sPESI or ECOG PS, and loss to follow-up within 30 days of PE diagnosis. Hemodynamic stability in the context of PE was defined by specific criteria excluding the conditions of hemodynamic instability. Patients were considered hemodynamically stable if they did not exhibit any signs of cardiac arrest, obstructive shock characterized by systolic blood pressure (BP) below 90 mmHg or the requirement for vasopressors to sustain BP at or above 90 mmHg, coupled with signs of end-organ hypoperfusion, or persistent hypotension indicated by a sustained systolic BP below 90 mmHg or a drop in systolic BP of 40 mmHg or more for longer than 15 minutes, not attributable to new-onset arrhythmia, hypovolemia, or sepsis [9].

Data collection and outcome measures

Patient characteristics and follow-up information were extracted from hospital records. Baseline variables examined included age, sex, underlying malignancies, heart failure, chronic lung disease, vital signs, and ECOG PS at the time of PE diagnosis, the imaging modality for definitive diagnosis, sPESI, onset of PE (in-hospital or out-of-hospital), initial treatment (anticoagulation and/or fibrinolytics), and post-PE management of unresectable or metastatic malignancies (chemotherapy or best supportive care (BSC)).

ECOG PS was determined based on each patient's daily self-care capabilities, with a score of 2 or lower assigned if patients spent more than 50% of the day moving, and a score of 3 or higher given if patients spent over 50% of the day in a bedridden or similar state [14].

The sPESI consists of six predictive variables, each accorded equal weight. These variables include age surpassing 80 years, a history of cancer, chronic cardiopulmonary disease, a pulse rate equal to or exceeding 110 beats per minute, systolic BP below 100 mmHg, and arterial oxyhemoglobin saturation less than 90%. Each individual variable contributes one point to the overall index score [2]. The cumulative point score for an individual patient is derived by summing these points, thereby categorizing each patient into one of two classes for the prediction of 30-day all-cause mortality: 0 points designating low risk, and ≥1 point indicating high risk. Samples with missing data were removed from further analysis. Right ventricular overload with right ventricular dilation (RVD), troponin, and brain natriuretic peptide (BNP) or N-terminal pro-brain natriuretic peptide (NT-proBNP) data were unavailable in the majority of patients (70.5%, 88.6%, and 61.4%, respectively) and were therefore omitted from analyses. The primary outcome measures in this study were 30-day all-cause mortality and overall survival (OS) post acute PE diagnosis.

Statistical analysis

Baseline characteristics were presented as mean and standard deviation (SD) for continuous data and counts and proportions for categorical data. Mann-Whitney U test and Fisher’s exact test were employed to compare clinical parameters between patients with ECOG PS ≤ 2 and those with ECOG PS ≥ 3, for continuous and categorical variables, respectively. Due to the limited number of events, logistic regression analyses for 30-day all-cause mortality, incorporating sPESI [1-4] and ECOG PS [14] as covariates, were not conducted. The relative risk for 30-day all-cause mortality between patients with ECOG PS ≤ 2 and ECOG PS ≥ 3 was calculated with zero-event adjustment by adding 0.5 to the number of events and 1.0 to the number in each group due to zero events in the ECOG PS ≤ 2 group [18,19]. The Kaplan-Meier method and log-rank test were utilized to evaluate OS differences between ECOG PS categories. Univariate and multivariate Cox regression models were applied to assess OS association with sPESI and ECOG PS. All statistical tests were two-tailed, with P < 0.05 considered statistically significant. R statistical software (version 4.3.1, R Foundation for Statistical Computing, Vienna, Austria) was used for analysis.

## Results

Patient selection flow and baseline characteristics

**Figure 1:**
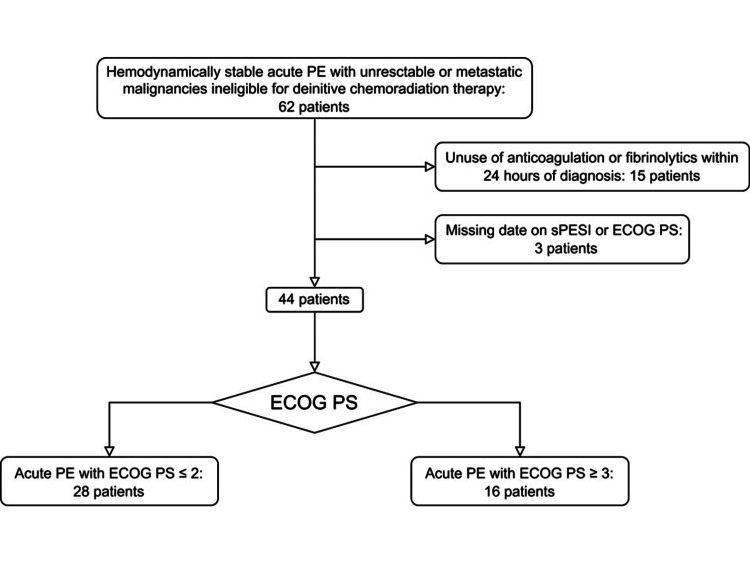
Patient selection flow. Graphical representation delineating the sequential stages involved in the identification, screening, and enrollment of patients from the initial cohort of potential participants. Abbreviations: ECOG PS, Eastern Cooperative Oncology Group Performance Status; PE, pulmonary embolism; sPESI, simplified Pulmonary Embolism Severity Index.

A total of 62 patients with unresectable or metastatic malignancies were identified as having hemodynamically stable acute PE through objective imaging tests during the investigation period (Figure 1). CTPA served as the definitive imaging modality for the entire cohort. Eighteen cases were subsequently excluded, consisting of 15 patients without anticoagulation or fibrinolytics within 24 hours after diagnosis and three patients with missing data on the sPESI or ECOG PS. The median duration of follow-up was 161 days (range (minimum-maximum): 8 to 1133 days), and none of the remaining 44 patients were lost to follow-up within 30 days of PE diagnosis. Thus, a total of 44 patients were included in the present analyses.

Age, sex, three comorbid illnesses (malignancy, heart failure, and chronic lung disease), vital signs at PE diagnosis, and the sPESI were comparable between patients with ECOG PS ≤ 2 and ECOG PS ≥ 3 (Table 1). The mean age was 68.5 years, with 62.3% of patients being female. Sixteen patients (36.4%) were classified as ECOG PS ≥ 3.

**Table 1:** Demographic and clinical characteristics. Comparison of patient characteristics between the acute pulmonary embolism (APE) group with ECOG PS ≤ 2 and the APE group with ECOG PS ≥ 3. Continuous variables are presented as mean and standard deviation, while categorical variables are shown as counts and percentages. Baseline characteristics were presented as mean and standard deviation (SD) for continuous data and counts and proportions for categorical data. Abbreviations: APE, acute pulmonary embolism; BP, blood pressure; CLD, chronic lung disease; ECOG PS, Eastern Cooperative Oncology Group Performance Status; HF, heart failure; sPESI, simplified Pulmonary Embolism Severity Index. ^†^ Defined as disorientation, lethargy, stupor, or coma.

Patient characteristics	APE with ECOG PS ≤ 2 (n = 28)	APE with ECOG PS ≥ 3 (n = 16)	p-value
Age	64.4 (13.5)	70.1 (10.9)	0.16
Sex (female)	14 (50.0％)	10 (62.5%)	0.53
Chronic comorbidities			
Malignancy	28 (100%)	16 (100%)	1.00
Heart failure (HF)	1 (3.6%)	2 (12.5%)	0.54
Chronic lung disease (CLD)	4 (14.3%)	1 (6.3%)	0.64
HF or CLD	4 (14.3%)	3 (18.8%)	0.69
Vital signs			
Heart rate ≥110	5 (17.9%)	2 (12.5%)	1.00
Systolic BP <100	1 (3.6%)	1 (6.3%)	1.00
Respiratory rate ≥30	1 (5.0%)	0 (0%)	1.00
O_2_ saturation <90% (on ambient air)	4 (14.3%)	2 (12.5%)	1.00
Altered mental status^†^	0 (0%)	0 (0%)	1.00
Temperature <36℃	3 (10.7%)	1 (6.3%)	1.00
sPESI ≥2	14 (50.0%)	7 (43.8%)	0.76
sPESI (continuous value)	1.6 (0.7)	1.6 (0.9)	0.83
Presentation setting (in-hospital occurrence)	13 (46.4%)	8 (50.0%)	1.00
Initial treatment			
Unfractionated heparin	14 (50.0%)	11 (68.8%)	0.34
Direct oral anticoagulants	13 (46.4%)	5 (31.3%)	0.36
Warfarin	0 (0%)	0 (0%)	1.00
Fondaparinux	1 (3.6%)	0 (0%)	1.00
Fibrinolytic agent	0 (0%)	0 (0%)	1.00
Post-pulmonary embolism management of malignancy			
Best supportive care	2 (7.1%)	8 (50.0%)	0.002
Chemotherapy	26 (92.9%)	8 (50.0%)	0.002

In comparison with patients with ECOG PS ≤ 2, those with ECOG PS ≥ 3 were more likely to be transitioned to BSC (7.1% vs. 50.0%; p= 0.002). However, there were no significant differences in the proportion of sPESI ≥ 2, in-hospital occurrence, and initial treatment medications between the two groups.

Variations in 30-day mortality rates by sPESI scores and ECOG PS

**Figure 2:**
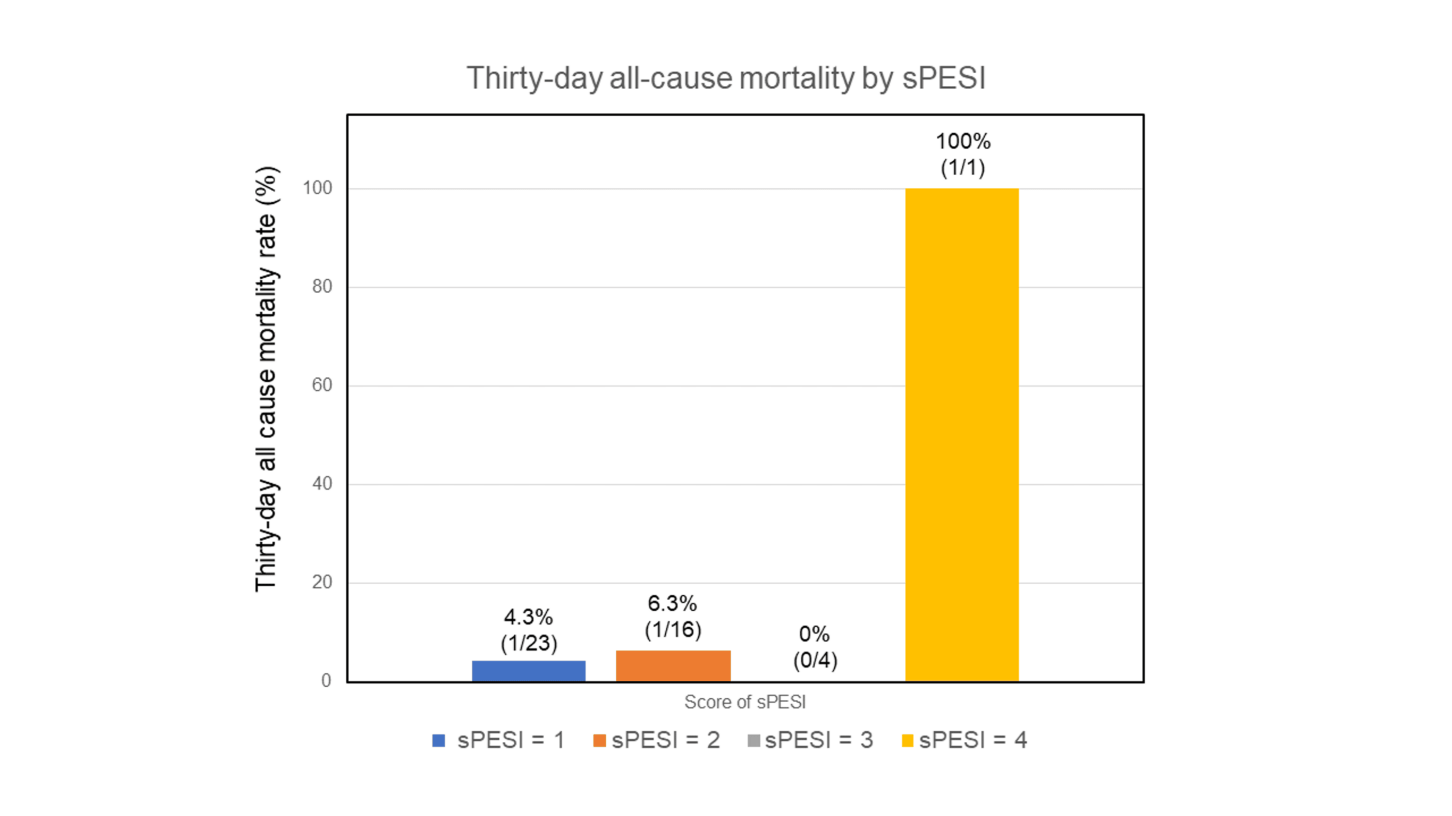
Thirty-day all-cause mortality by sPESI. Stratified comparison of 30-day all-cause mortality based on simplified Pulmonary Embolism Severity Index (sPESI) in patients diagnosed with acute pulmonary embolism.

The 30-day all-cause mortality rates, stratified by sPESI scores, were 4.3% (one out of 23) for a score of 1 point, 6.3% (one out of 16) for a score of 2 points, 0% (0 out of four) for a score of 3 points, and 100% (one out of one) for a score of 4 points. However, the limited number of events precluded drawing meaningful statistical conclusions between these groups (Figure 2).

**Figure 3:**
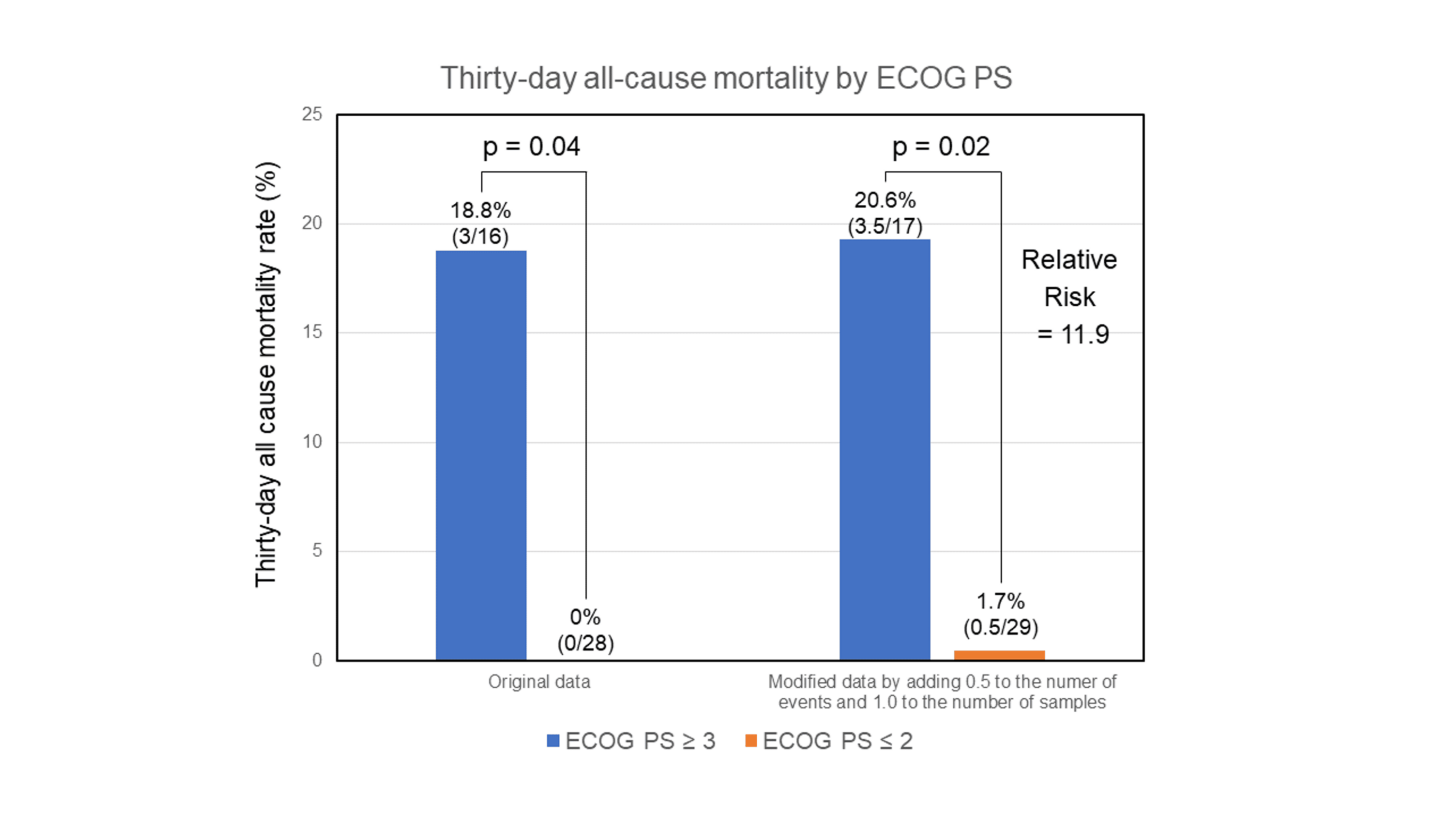
Thirty-day all-cause mortality by ECOG PS. Comparison of 30-day all-cause mortality rates in the acute pulmonary embolism (APE) subgroup with Eastern Cooperative Oncology Group Performance Status (ECOG PS) ≤ 2 and the APE subgroup with ECOG PS ≥ 3. The relative risk of 30-day all-cause mortality between the two groups was calculated using adjusted data, with 0.5 added to the number of events and 1.0 added to the number of samples.

Over the 30-day follow-up period, three patients (18.8%) out of 16 with ECOG PS ≥ 3 died, while none of the 28 patients with ECOG PS ≤ 2 succumbed (p = 0.04). With a zero-event adjustment, the relative risk for mortality in patients with ECOG PS ≥ 3 was found to be 11.9-fold higher compared to their counterparts with ECOG PS ≤ 2. This analysis underscores a significantly elevated risk of mortality within a 30-day period for patients presenting with a more advanced ECOG PS (Figure 3).

The direct causes of death included type 1 respiratory failure (one case), brainstem infarction due to disseminated intravascular coagulation (DIC) (one case), and severe dehydration and malnutrition due to cachexia (one case) (Table 2). Notably, no fatal cases resulting from hemodynamic instability followed by circulatory failure were reported.

**Table 2:** Patient characteristics of three fatal cases within 30 days after diagnosis of acute pulmonary embolism. Comparative analysis of patient characteristics within the study cohorts, focusing on three fatal cases occurring within 30 days following the diagnosis of acute pulmonary embolism. Abbreviation: BSC, best supportive care; DIC, disseminated intravascular coagulation; PS, Performance Status; sPESI, simplified Pulmonary Embolism Severity Index.

Age	Sex	Type of malignancy	Direct cause of death	PS	sPESI	Presentation setting	Management of malignancy
79	Male	Lung adenocarcinoma	Brainstem infarction due to DIC	4	2	Inpatient	BSC
86	Female	Gastric adenocarcinoma	Type 1 respiratory failure	4	4	Outpatient	BSC
72	Male	Gastric adenocarcinoma	Severe dehydration and malnutrition due to cachexia	3	1	Outpatient	Chemotherapy

Impact of ECOG PS on the long-term prognosis and its independence from the sPESI

**Figure 4:**
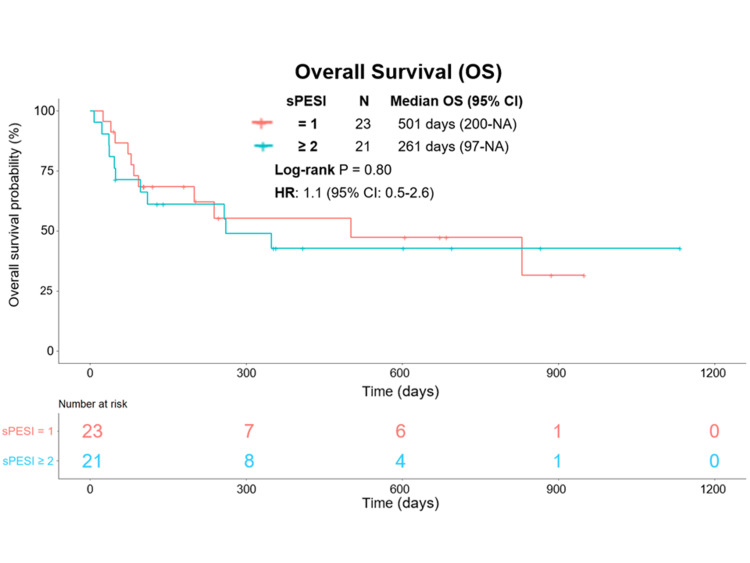
Kaplan-Meier estimates of overall survival by sPESI. Comparative analysis of Kaplan-Meier estimates depicting overall survival in distinct subgroups of acute pulmonary embolism (APE) based on simplified Pulmonary Embolism Severity Index (sPESI), specifically contrasting the APE subgroup with a sPESI score = 1 against the APE subgroup with a sPESI score ≥ 2. Abbreviations: CI, confidence interval; OS, overall survival; PE, pulmonary embolism.

**Figure 5:**
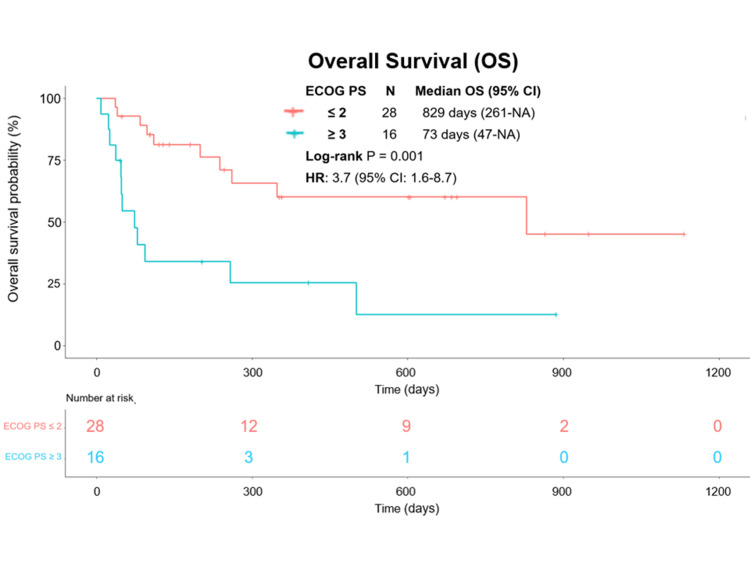
Kaplan-Meier estimates of overall survival by ECOG PS. Comparative analysis of Kaplan-Meier estimates depicting overall survival in distinct subgroups of acute pulmonary embolism (APE) based on Eastern Cooperative Oncology Group Performance Status (ECOG PS), specifically contrasting the APE subgroup with ECOG PS ≤ 2 against the APE subgroup with ECOG PS ≥ 3. Abbreviations: CI, confidence interval; OS, overall survival; PE, pulmonary embolism; sPESI, simplified Pulmonary Embolism Severity Index.

During the median follow-up duration of 161 days, 22 patients succumbed. No statistically significant disparity in median overall survival (OS) was noted between the patient cohorts with sPESI = 1 and those with sPESI ≥ 2 (Figure 4). Conversely, the median OS exhibited a significantly shorter duration in the ECOG PS ≥ 3 group compared to the ECOG PS ≤ 2 group (829 days vs. 73 days; p = 0.001) (Figure 5). ECOG PS ≥ 3 demonstrated an independent association with an incremented hazard of OS in both univariate and multivariate Cox regression models (unadjusted hazard ratio (HR), 3.7; 95% confidence interval (CI), 1.6 to 8.7; p = 0.002; adjusted HR, 4.0; 95% CI, 1.7 to 9.4; p = 0.002) (Table 3).

**Table 3:** Univariate and multivariate Cox regression analyses for overall survival. Cox regression analyses conducted to assess overall survival in relation to prognostic factors associated with ECOG PS ≥ 3 and sPESI. Abbreviation: CI, confidence interval; ECOG PS, Eastern Cooperative Oncology Group Performance Status; sPESI, simplified Pulmonary Embolism Severity Index.

Predictors	Univariate		Multivariate	
	Hazard ratio (95% CI)	p-value	Hazard ratio (95% CI)	p-value
ECOG PS ≥ 3	3.7 (1.6-8.7)	0.002	4.0 (1.7-9.4)	0.002
sPESI ≥ 2	1.1 (0.5-2.6)	0.77	1.4 (0.6-3.4)	0.43

## Discussion

In recent years, the management paradigm for acute PE has shifted toward outpatient care, propelled by the emergence of DOACs and approaches for delineating cohorts at low risk for short-term mortality, exemplified by the sPESI [2-4,7-9]. However, an established method for accurately stratifying the risk of PE cases with complicating factors, such as cancer, remains elusive.

Advancements in chemotherapy and supportive care have significantly improved survival rates, with lots of malignant tumors now exhibiting prolonged survival. As the understanding of medical conditions in cancer patients has become increasingly intricate, it holds considerable clinical significance to provide clear and concise evidence regarding the appropriateness of outpatient anticoagulation therapy for hemodynamically stable PE in patients with unresectable or metastatic cancer, especially for non-oncologists increasingly encountering cancer-related acute conditions.

**Figure 6:**
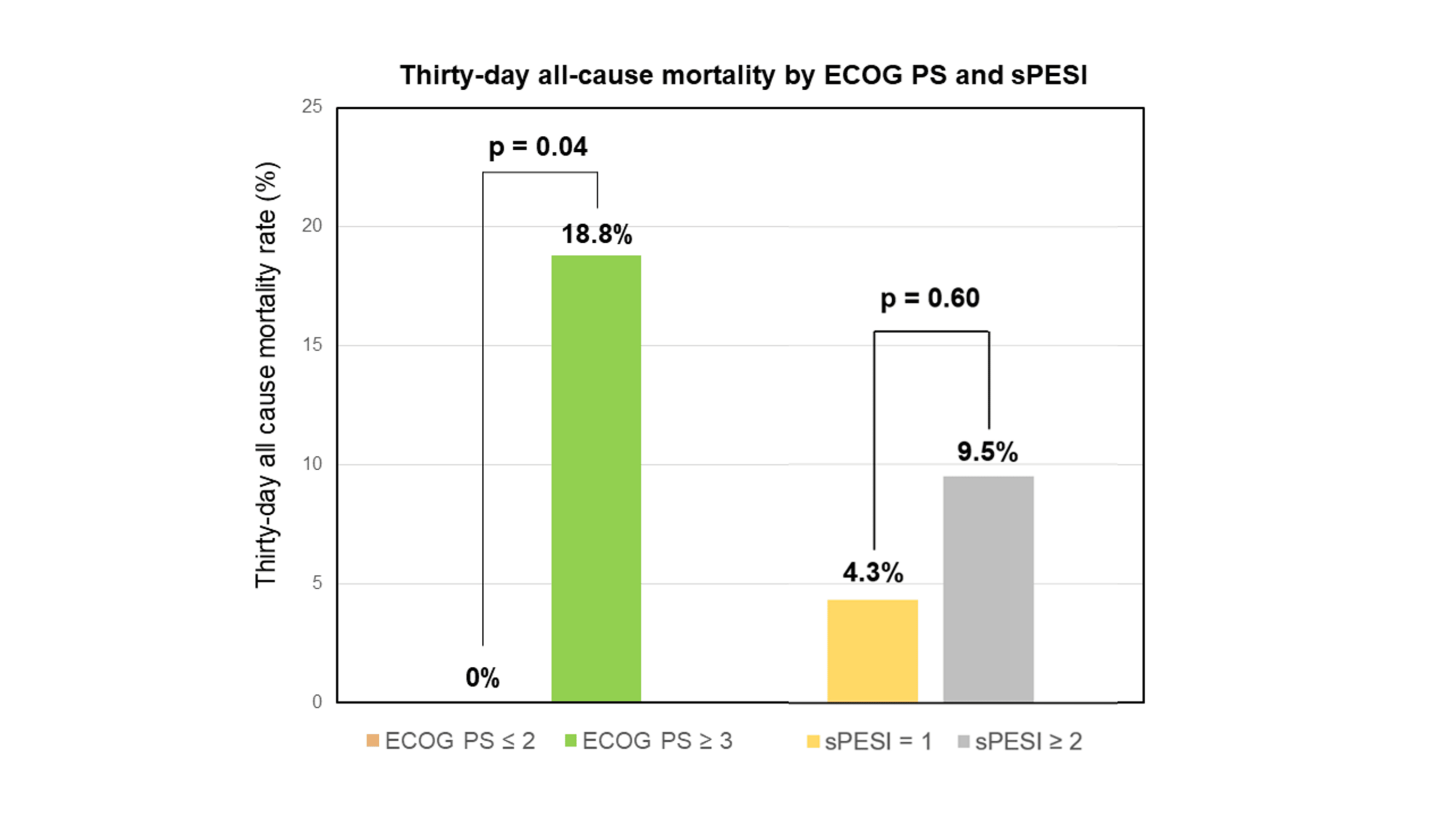
Thirty-day all-cause mortality by ECOG PS and sPESI. Stratified comparison of 30-day all-cause mortality of acute pulmonary embolism based on Eastern Cooperative Oncology Group Performance Status (ECOG PS) and simplified Pulmonary Embolism Severity Index (sPESI) between patients with ECOG PS ≤ 2 and those with ECOG PS ≥ 3, as well as between patients with sPESI = 1 and sPESI ≥ 2, respectively.

This retrospective study delves into the influence of ECOG PS on the prognosis of hemodynamically stable acute PE in the presence of concomitant unresectable or metastatic malignant tumors. It also explores the independence of ECOG PS from the sPESI. Key findings include: (1) ECOG PS ≥ 3 was associated with an adverse prognosis for both 30-day all-cause mortality and OS; (2) ECOG PS ≥ 3 stood as an independent predictive factor for OS, irrespective of the sPESI; (3) an instance of short-term mortality within 30 days post-PE diagnosis was observed even with an sPESI score of 1, whereas no fatal cases occurred in individuals with ECOG PS ≤ 2 (Figure 6).

ECOG PS serves as a key assessment tool for evaluating functional capacity, commonly employed in devising treatment strategies for advanced cancer patients. Moreover, we intend to delve into the consideration of cutoff settings for ECOG PS as a determinant of short-term mortality risk in hemodynamically stable PE among individuals with unresectable or metastatic malignancies. Traditionally, PE has been predominantly linked with mortality resulting from circulatory failure due to progressive obstructive shock, apart from treatment-related deaths due to anticoagulant therapy, prompting the early initiation of anticoagulation therapy for prognostic improvement [9]. However, in cases of hemodynamically stable PE, alternative causes other than obstructive shock have gained significance. From our current investigation and prior studies, it is apparent that, alongside circulatory failure, additional mortality factors include respiratory failure, dehydration and malnutrition, and exacerbation of background diseases such as malignancy [20-22].

Given that PE impacts both the pulmonary artery and respiratory systems, initiation of anticoagulation therapy in hemodynamically stable patients may still pose risks if there is a pre-existing respiratory compromise or a history of lung disease. The respiratory burden imposed by PE may exceed an individual's respiratory reserve capacity, potentially culminating in fatal respiratory failure [20,21]. Furthermore, acute conditions, exemplified by PE, can precipitate loss of muscle skeletal mass, resulting in the risk not only for physical impairment but also for compromised swallowing function [23]. For patients with pre-existing swallowing dysfunction, sustaining oral intake may become challenging, leading to dehydration and malnutrition, even post-successful PE treatment, culminating in a terminal condition. In these instances, ECOG PS ≥ 3 could be considered a prognostic indicator due to respiratory and swallowing muscle weakness [24-26].

Thirdly, the underlying disease of PE, notably advanced cancer and exacerbated DIC, warrants consideration. Managing cancer progression is frequently required to improve coagulation status on these occasions. However, as ECOG PS deteriorates to 3 or higher, the feasibility of curative interventions, including surgery or chemoradiation, and the effectiveness of palliative chemotherapy are compromised due to tolerability concerns. Consequently, transitioning to BSC becomes inevitable in the majority of cases, often resulting in a shortened timeline toward a fatal outcome. Therefore, we have opted for ECOG PS ≥ 3 as the cutoff, considering the substantial reduction in the likelihood of cancer cachexia improvement beyond this threshold.

Assessment of ECOG PS is readily executable in the clinical setting, akin to the sPESI, through patient interviews and physical examinations evaluating walking status [14]. While prior studies underscored its utility as a predictive factor for acute PE in the context of malignancies [11,17], this study distinguishes itself by demonstrating the independence of ECOG PS ≥ 3 from the sPESI as a long-term prognostic factor for acute PE in patients with unresectable or metastatic malignant tumors.

The prognostic variables comprising the PESI were derived from survival analyses conducted in the ICOPER study, a multicenter retrospective cohort involving over 10,000 inpatient cases [1,6]. The subsequent development of the simplified version, the sPESI model, focused on six variables from the PESI model that demonstrated statistical significance in another cohort of over 900 outpatient cases [2]. Although both PESI and sPESI serve as prognostic indicators for short-term outcomes, with 30-day all-cause mortality as the outcome measure, the sPESI failed to exhibit statistical significance as a predictive indicator for OS in this investigation. This may be attributed to the exclusion of cases with hemodynamic instability, which were included in the original study, and the limited number of cases leading to insufficient statistical power.

Conversely, ECOG PS demonstrated statistical significance as a prognostic factor for OS in multivariate analysis with the sPESI as a covariate. These findings imply that ECOG PS holds the potential as a more efficacious indicator than the sPESI for predicting OS in patients with hemodynamically stable acute PE and unresectable or metastatic malignancies. Additionally, the study suggests that the medium to long-term prognosis may be more reliant on the status of malignancies and their treatment conditions than on PE itself in these patients. This is evidenced by the higher frequency of BSC as the post-PE management choice for malignancies in patients with ECOG PS ≥ 3.

Furthermore, the study revealed a subtle difference in 30-day all-cause mortality between patients with an sPESI score of 1 and those with a score of 2 (4.3% vs. 6.3%, respectively). Short-term mortality in patients with an sPESI score of 1 due to malignancies was comparable to that reported in the COMMAND VTE Registry [13]. Although the sPESI score was formulated to identify outpatient-treatable low-risk groups for short-term mortality, with a score of 0 serving as the reference standard [2-4], short-term mortality in these patients was clinically significantly higher compared to the traditional low-risk group of acute PE [1-3]. This underscores the challenge of clearly categorizing the short-term mortality of acute PE as low risk using the sPESI alone in cancer patients.

In contrast, a significant contrast in 30-day all-cause mortality was observed, ranging from 0% in patients with ECOG PS ≤ 2 to 18.8% in those with ECOG PS ≥ 3. The prognosis in patients with ECOG PS ≤ 2 aligns with that of a low-risk group in acute PE [1-4], suggesting that outpatient treatment of acute PE with incurable malignancies and ECOG PS ≤ 2 may be a feasible approach. On the contrary, the prognosis in patients with ECOG PS ≥ 3 indicates a short-term mortality risk comparable to that of hemodynamically unstable PE [5]. This finding is crucial for facilitating informed discussions with PE patients about their medical condition and management.

Due to the limited number of events in this study, logistic analyses for 30-day all-cause mortality were unfeasible. Consequently, the independence of ECOG PS from the sPESI concerning short-term mortality could not be confirmed. However, despite the absence of significant differences in major confounding factors such as the sPESI, location of onset, and initial treatment between ECOG PS ≤ 2 and ECOG PS ≥ 3 groups, a clinically significant difference in short-term mortality was observed between the two groups.

Considering the lack of significant differences in short-term mortality between sPESI = 1 and ≥ 2 groups, ECOG PS is also anticipated to be a promising prognostic factor for short-term mortality in acute PE with unresectable or metastatic malignancies (Figure 6). Additionally, since this study focuses on cases of unresectable or metastatic malignant tumors, the favorable prognosis observed in PE patients with advanced malignant tumors and ECOG PS ≤ 2 may be extrapolated to those with more favorable and stable malignancies, such as early-stage or resectable malignancies. To validate whether the combination of ECOG PS with the sPESI enhances the predictive capability for the short-term prognosis of acute PE with malignancies, further validation in a larger cohort is warranted.

Study limitations

This investigation is subject to several inherent limitations. Firstly, being a single-center retrospective study with initial treatment decisions left to the discretion of individual physicians, potential concerns arise regarding data gaps, incomplete follow-up, as well as the possibility of selection bias and unmeasured confounding factors. Notwithstanding, data aggregation gaps, aside from right ventricular overload, were minimal. The examination encompassed a diverse array of malignant tumors across organs, exhibiting no apparent bias in primary organs of malignancy and pathological diagnoses between the two groups (see Appendix).

Secondly, the literature extensively establishes the significance of right ventricular overload as a precursor to the progression from right ventricular failure to obstructive shock in acute PE patients [9,27-30]. It stands as a crucial short-term prognostic factor, complementing hemodynamic instability and the sPESI [9]. However, in this study, crucial data such as echocardiographic findings, troponin levels, and BNP or NT-proBNP were often unmeasured. Consequently, the potential confounding effect of right ventricular overload on both ECOG PS and short-term prognoses of acute PE in the presence of unresectable or metastatic malignant tumors could not be verified. Notably, none of the reported 30-day all-cause mortality cases in this cohort were attributed to circulatory failure resulting from obstructive shock after the acute PE diagnosis, suggesting a limited impact of right ventricular overload as a confounding factor within this cohort.

Thirdly, the inclusion of both inpatient and outpatient cases in this study to assess the prognostic utility of ECOG PS might limit specificity for outpatient treatment. Therefore, validation within a larger cohort exclusively composed of outpatient cases is desirable.

Lastly, while our study utilized imaging diagnostics to select patients with acute PE, it is important to acknowledge the potential inclusion of pulmonary tumor embolism (PTE) cases. Differentiating between acute PE and PTE typically requires pathological examination, which was not feasible for surviving patients and was not performed in deceased patients within this cohort. Therefore, we cannot definitively exclude the presence of PTE among our study population, representing a limitation of this research.

## Conclusions

The presence of ECOG PS ≥ 3 emerged as an independent predictor associated with an unfavorable long-term prognosis in hemodynamically stable acute PE patients with unresectable or metastatic malignancies. Conversely, ECOG PS ≤ 2 exhibited a concurrent association with a favorable short-term prognosis. To validate and enhance generalizability, further prospective studies with larger cohorts are imperative. Such investigations should specifically focus on evaluating the safety and efficacy of outpatient anticoagulation therapy in this patient population.

## Appendices

**Table 4:** Primary organ of malignancy. Comparison of the primary organ affected by malignancies between the acute pulmonary embolism (APE) group with ECOG PS ≤ 2 and the APE group with ECOG PS ≥ 3. Abbreviations: APE, acute pulmonary embolism; DLBCL, diffuse large B-cell lymphoma; ECOG PS, Eastern Cooperative Oncology Group Performance Status; HCC, hepatocellular carcinoma; MM, multiple myeloma; NSCLC, non-small cell lung cancer.

	APE with ECOG PS ≤ 2 (n = 28)	APE with ECOG PS ≥ 3 (n = 16)
Primary organ of malignancy	Colorectal cancer: 6 cases. Endometrial cancer: 3 cases. Gastric cancer: 3 cases. NSCLC: 3 cases. Ovarian cancer: 3 cases. Breast cancer: 2 cases. DLBCL: 1 case. HCC: 1 case. MM: 1 case. Cervical cancer: 1 case. Cholangiocarcinoma: 1 case. Prostate cancer: 1 case. Uterus leiomyosarcoma: 1 case. Retroperitoneum dedifferentiated liposarcoma: 1 case.	Gastric cancer: 3 cases. NSCLC: 3 cases. Pancreatic cancer: 3 cases. Colorectal cancer: 2 cases. Breast cancer: 1 case. Cervical cancer: 1 case. Thyroid cancer: 1 case. Ovarian cancer: 1 case. Unknown origin: 1 case.

**Table 5:** Pathological diagnosis of malignancy. Comparison of the pathological diagnosis of malignancies between the acute pulmonary embolism (APE) group with ECOG PS ≤ 2 and the APE group with ECOG PS ≥ 3. Abbreviations: APE, acute pulmonary embolism; DLBCL, diffuse large B-cell lymphoma; ECOG PS, Eastern Cooperative Oncology Group Performance Status; HCC, hepatocellular carcinoma; MM, multiple myeloma.

	APE with ECOG PS ≤ 2 (n = 28)	APE with ECOG PS ≥ 3 (n = 16)
Pathological diagnosis of malignancy	Adenocarcinoma: 12 cases. Serous carcinoma: 2 cases. No data: 2 cases (HCC and prostate cancer). Carcinosarcoma: 1 case. Clear cell adenosquamous carcinoma: 1 case. De-differentiated liposarcoma: 1 case. DLBCL: 1 case. MM: 1 case. Invasive ductal carcinoma: 1 case. Metaplastic carcinoma: 1 case. Leiomyosarcoma: 1 case. Endometrioid carcinoma: 1 case. Epithelial carcinoma: 1 case. Serous adenocarcinoma: 1 case. Signet ring cell carcinoma: 1 case.	Adenocarcinoma: 11 cases. Carcinoma: 1 case. Follicular carcinoma: 1 case. Mucinous adenocarcinoma: 1 case. Serous carcinoma: 1 case. No data: 1 case (pancreatic cancer).
